# Advances in highly active one-carbon metabolism in cancer diagnosis, treatment and drug resistance: A systematic review

**DOI:** 10.3389/fonc.2022.1063305

**Published:** 2022-12-01

**Authors:** Shuang Liu, Zheng-Miao Wang, Dong-Mei Lv, Yi-Xuan Zhao

**Affiliations:** Department of Ultrasound, The Second Hospital of Jilin University, Changchun, China

**Keywords:** chemotherapy, folate cycle, randomized-controlled trials, DNA methylation, methionine cycle

## Abstract

**Study background objectives:**

Cancer poses a significant health concern as it is incurable. Every year, research on how to treat and eradicate this chronic condition is done. This systematic review will unmask the recent developments concerning highly active 1C metabolism with regard to cancer diagnosis, treatment, and drug resistance. The significance of this study is rolling out evidence-based evidence on the importance of one-carbon metabolism in cancer diagnosis and treatment.

**Methods:**

Eight randomized controlled trials (RCTs) were reviewed from five electronic databases – EMBASE, Scopus Review, Google Scholar, Web of Science, and PubMed. Outcomes from the eight studies were analyzed to paint a picture of the topic in question. While the Preferred Reporting Items for Systematic Reviews and Meta-Analysis’ (PRISMA) protocol guided the initial literature search, The Grading of Recommendations Assessment, Development, and Evaluation (GRADE) approach informed quality assessments of the eligible studies.

**Conclusion:**

Since its emergence in the 1980s, 1C metabolism has been investigated and broadened to capture essential aspects of cancer treatment, diagnosis, and drug resistance. The review found that metabolites like folic acid could be used to detect different types of cancer. The metabolic pathways could induce tumorigenesis and DNA methylation, hence drug resistance.

**Systematic review registration:**

https://inplasy.com/projects/, identifier INPLASY2022110099.

## Introduction

One-carbon (IC) metabolism is characterized by a series of interconnected biological processes, like folate and methionine cycles, that play key roles in cellular functions and provide one carbon unites, or rather, the methyl groups, for the amino acids, deoxyribonucleic acid (DNA), phospholipid and creatine synthesis (1). Better yet, cells need one-carbon units for methylation, reductive metabolism, and nucleotide synthesis, biological processes which anchor high rates of proliferation of cancer cells. Consequently, medications targeting one-carbon metabolism and anti-folates have been used in cancer treatment.

Researchers have shifted their attention in the last decades toward one-carbon metabolism since the pathway involves crucial steps like DNA synthesis, where the metabolic process is reprogrammed. This alteration plays a crucial role in the diagnosis and therapy of cancers. The altered metabolism informs the unregulated and rapid proliferation of cancerous cells, which necessitates an enormous supply of nucleotides.

**Figure 1:**
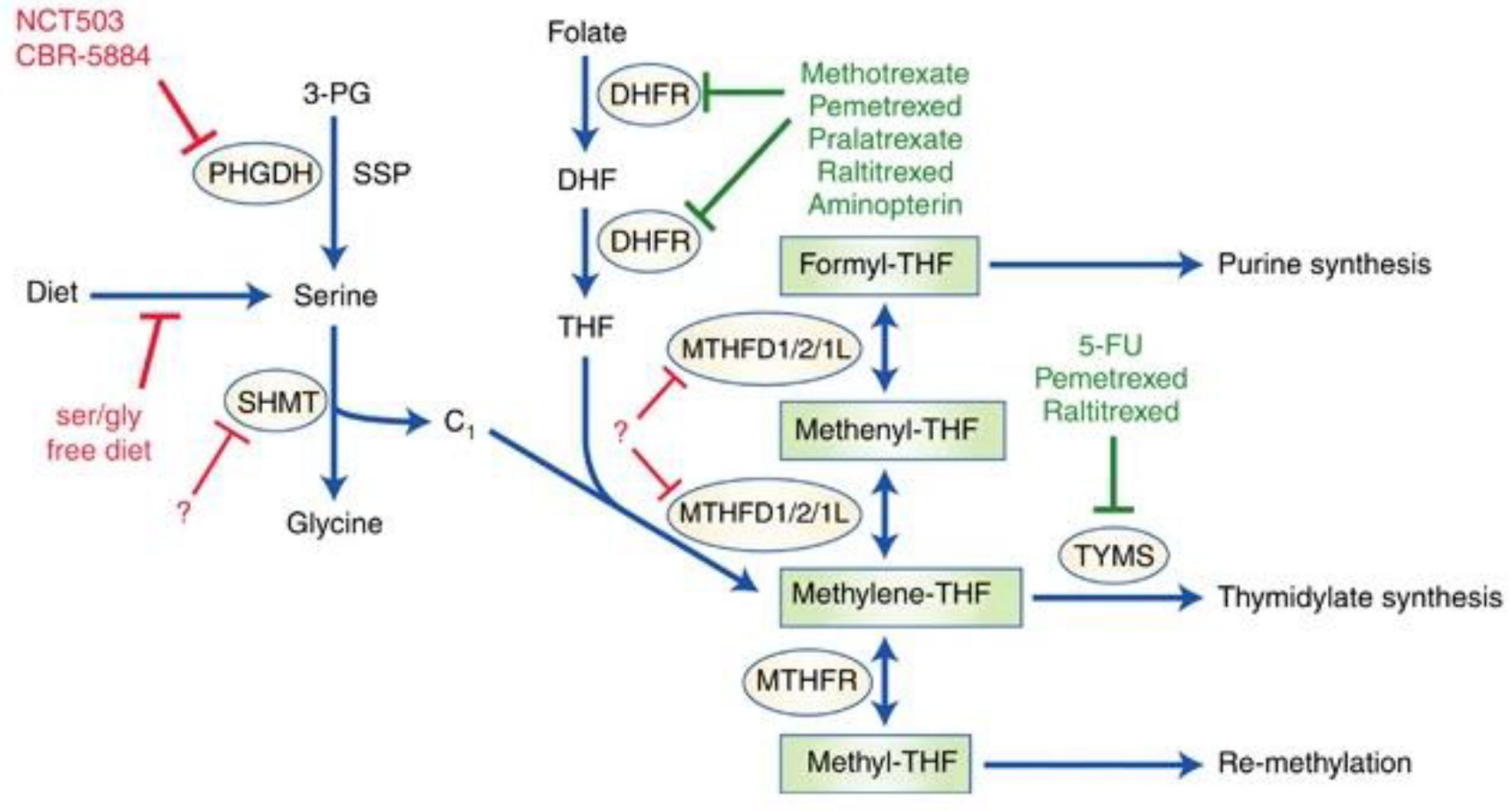
*One-Carbon Metabolism in Cancer. Note.* While one-carbon metabolic pathways characterized by solid chemotherapeutics are highlighted in green, potential future targets are highlighted in red. The solid red line represents interventions under study. Dashed lines represent potential targets that could be investigated. Enzymes regulating these biological pathways are circles. Serine, which can also be synthesized *de novo* from 3-PG *via* SSP, a glycolytic intermediate, where PHGDH stands out as the prominent enzyme. Dietary folate is converted to DHF by DHFR and thereafter to THF, which is a recipient of a single carbon. SHMT1/2 catabolizes serine to glycine and subsequently yields a single carbon unit, which THF accepts, hence the formation of methylene-THF. MTHFD1/2/1L can convert the latter to formyl-THF through the intermediate, methenyl-THF. The formyl-THF gives out its single carbon to the purine synthesis cascade. The methylene-THF can either be converted to methyl-THF by MTHFR or give out its single carbon unit to the thymidylate synthesis cascade. The methyl-THF supply carbon units for processes where methionine is recycled.

**Figure 1** illustrates advances concerning highly active one-carbon metabolism and possible future therapeutics for cancer targeting the pathway.

**Figure 2:**
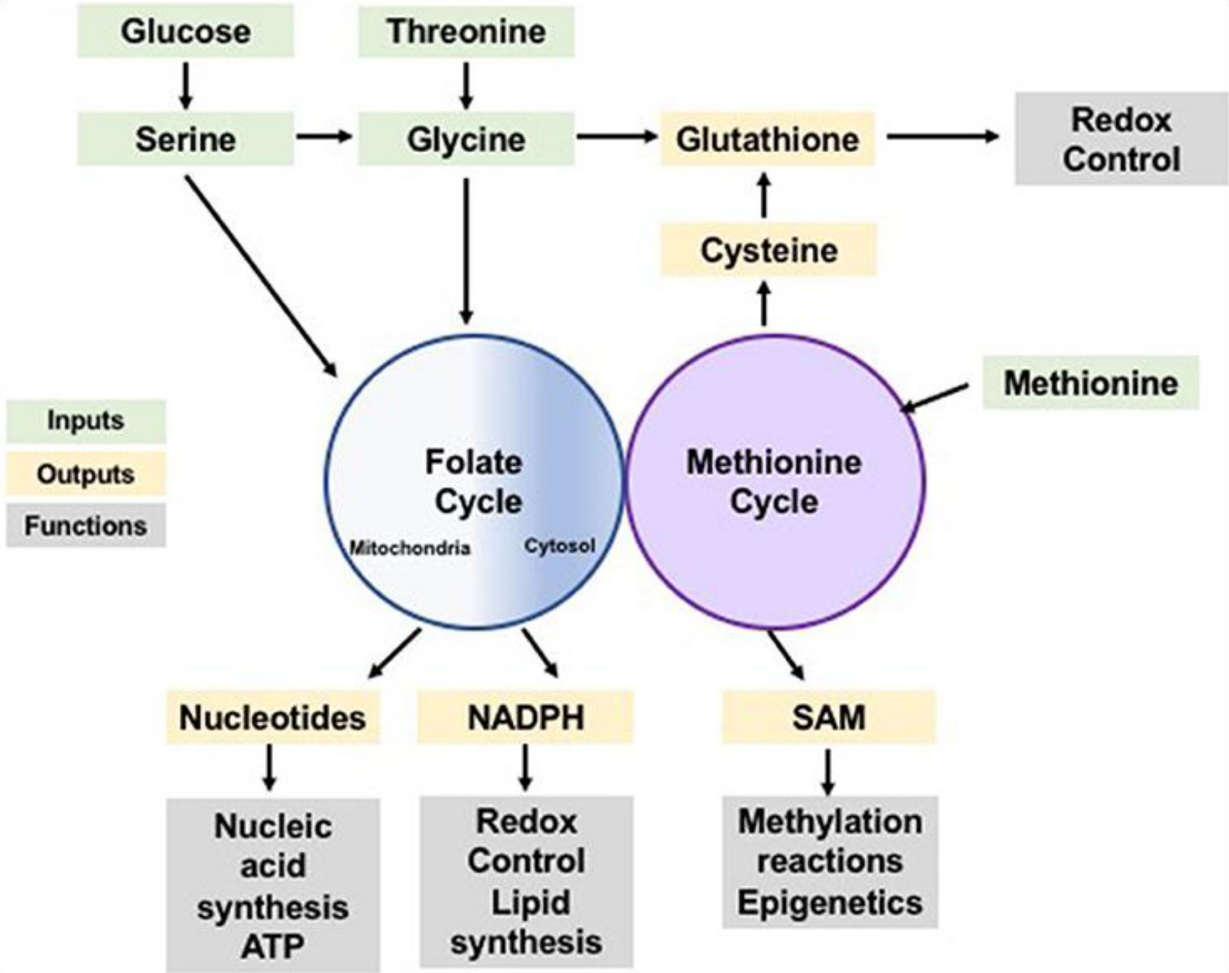
*One-Carbon Metabolism is a Normal Cellular Activity of Integrating Nutrients and Availability. Note.* Amino acids and glucose are inputs of the methionine and folate cycles (represented by the green color) which contribute one-carbon units that can be utilized in the anabolic synthesis of various building blocks and co-factors (represented by the yellow color) alongside reducing species. The products of these processes perform various biological functions (represented by the grey color), including biosynthesis of biomolecules, modulation of reduction-oxidation reaction, sustaining homeostasis within the cells, and post-translational modification.

One-carbon units are used in two biological pathways: the methionine cycle and the folate cycle (see **Figure 2**). In the folate pathway, dihydrofolate reductase (DHFR) reduces folic acid to tetrahydrofolate (THF), a biologically inactive compound (2). In this reduced state, glycine, and serine one-carbon units become transferrable by the glycine decarboxylase (GLDC), a component of the glycine cleavage system [GCS], and serine hydroxymethyltransferase (SHMT), respectively, and this proceeds to the formation of methyl-THF by the THF. Upon methylation, THF is set t undergo multiple redox reactions catalyzed by the polyfunctional enzyme methylenetetrahydrofolate dehydrogenase (MTHFD1/2/1L), with mitochondrial and cytosolic isoforms (3, p. 257), (4, p. 1, 149). S-adenosyl-methionine demethylation produced S-adenosyl-homocysteine (SAH), which is subsequently transformed to homocysteine, characterizing the end of the cycle. The crosstalk existing between methionine and folate cycles spans beyond homocysteine re-methylation, as the *de novo* adenosyl triphosphate synthesis is powered by the folate cycle and is directly involved in SAM formation. SAM is a crucial donor of various multiple reactions (5). Further, the *de novo* adenosine triphosphate generation *via* the folate cycle is somewhat indispensable in maintaining energetic homeostasis, especially when energy is demanded in large quantities, like during the proliferation of cells at high rates.

Advances in highly active 1C metabolism have diverted to dietary aspects where vitamin B12 and folate have been studied. Some of these studies posit that high concentrations of folate in the blood could enhance methylation reactions of epigenetics (**Figure 2**), thereby causing cancer resistance and incidence (6, 7). This can be an effective intervention and equally a diagnostic measure. On the one hand, the use of anti-folate could be an effective treatment measure as it will eliminate the carcinogenic functions and biological actions of the folate in the blood. As with diagnosis, the onset of cancer or the mutation can be detected by a certain concentration of folate in the serum, whereby vitamin B12 has been found to be 300 pmol/L (7).

**Figure 3:**
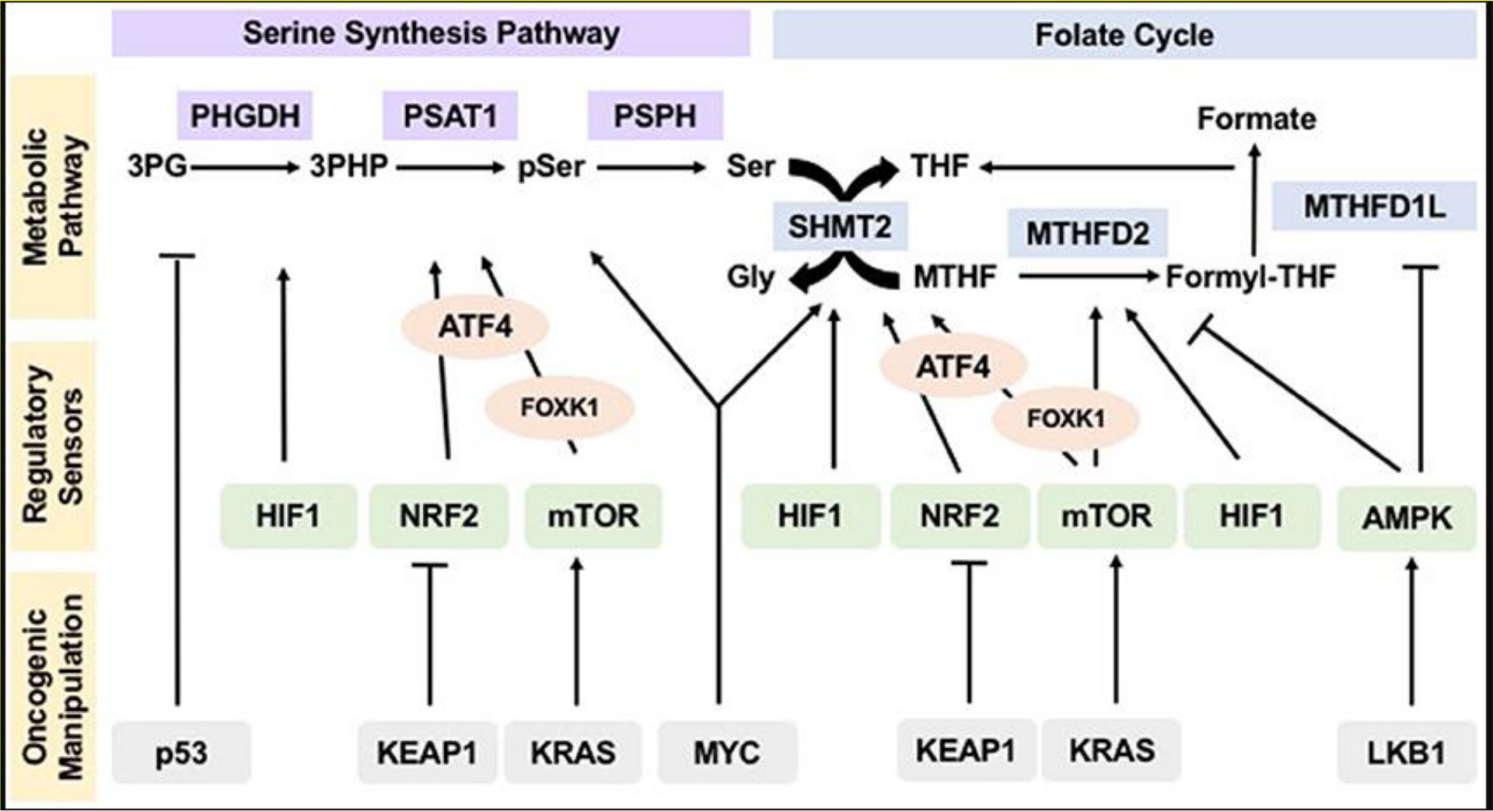
*Oncogenic Manipulation, Regulatory Sensor, and Metabolic Pathways of the Serine Synthesis Pathway and Folate Cycle. Note.* Sensors of nutrients and energy levels, AMP-activated protein kinase, rapamycin’s mammalian target, and reduction-oxidation potential sensors, NRF2 and HIF1, regulate various metabolic pathways. KEAP1, LKB1, tumor suppressor p53 KRAS, and the MYC modulate regulatory sensors, influencing flux and operation pathways within the metabolic system. This allows hyperactivity which sustains tumorigenesis, proliferation, and the subsequent mutation or resistance to treatment.

Evidently, the one-carbon metabolism pathway is a tightly regulated biological process. **Figure 3** shows how tumor and oncogenesis suppressors influence the regulation of the one-carbon metabolic pathway, which drives tumorigenesis. The pathway explains how cancer resistance can result and how the treatment can be achieved.

NRF2: nuclear factor erythroid-derived 2

HIF1: hypoxia included factor 1

KEAP1: kelch-like ECH-associated protein 1

KRAS: Kirsten rat sacroma

MYC: myelocytomatosis

Cancer cells depend on 1-carbon units for methylation, nucleotide synthesis, and reductive metabolism. These pathways anchor high proliferative rates in affected cells. 1-carbon metabolism’s byproducts, mainly folates, are responsible for cancer development. 

Even though the current literature defines the relationship between 1-carbon metabolism and cancers, as stated above, not much has been reported about the advances in highly active 1C metabolism and its outcomes in cancer diagnosis, treatment, and drug resistance. This review will explore these aspects through randomized controlled trials.

## Materials and methods

### Study design and searched databases

The initial literature search was performed in five electronic databases (EMBASE, Web of Science, PubMed, Google Scholar, and Scopus Review) under the provisions of the Preferred Reporting Items for Systematic Reviews and Meta-Analysis’ (PRISMA) (8, 9). An initial literature search was a procedural process where studies were identified through titles and abstracts, after which they would be subjected to full-text analysis.

### Search strategy

**Figure 4:**
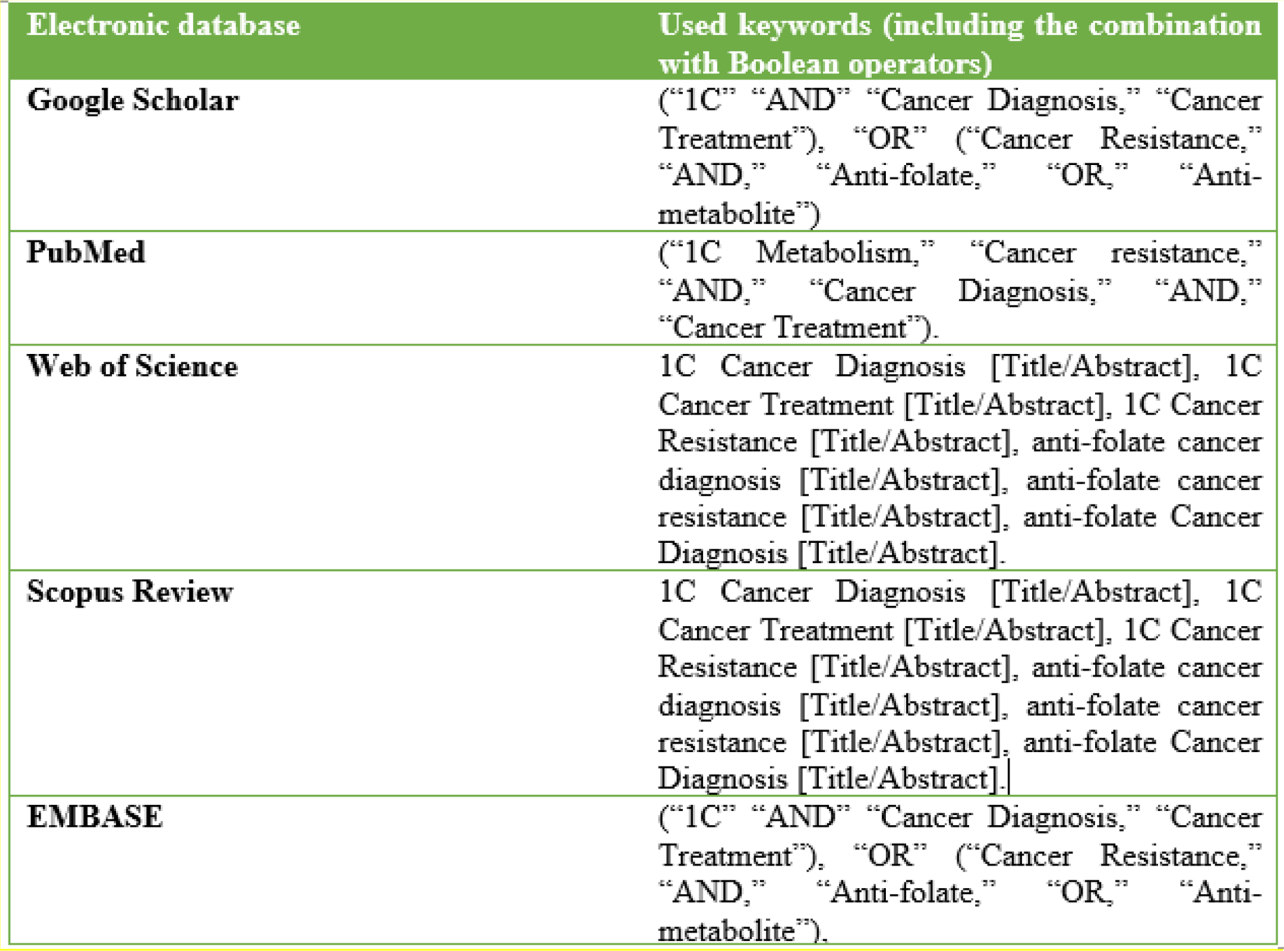
An Illustration of the search Strategy Showing the Electronic Databases where Literature search was performed against used Keywords/Search terms.

The initial literature search was performed based on the PRISMA protocol on the above-mentioned electronic databases (EMBASE, Web of Science, PubMed, Google Scholar, and Scopus Review) (**Figure 4**). Boolean operators “AND” and “OR” guided the initial literature search, as they were combined with the keywords pertinent to the topic. The keywords and Boolean operators were used hand-in-hand to yield potential studies. Of note, the Boolean operator “OR” linked similarly significant keywords. The Boolean operator “AND” connected dissimilar keywords. The following combination of keywords and Boolean operators were used: (“1C,” “AND” “Cancer Diagnosis,” “Cancer Treatment”), “OR” (“Cancer Resistance,” “AND,” “Anti-folate,” “OR,” “Anti-metabolite”), (“1C Metabolism,” “Cancer resistance,” “AND,” “Cancer Diagnosis,” “AND,” “Cancer Treatment”). Also, title and abstract screening were used to identify studies: 1C Cancer Diagnosis [Title/Abstract], 1C Cancer Treatment [Title/Abstract], 1C Cancer Resistance [Title/Abstract], anti-folate cancer diagnosis [Title/Abstract], anti-folate cancer resistance [Title/Abstract], and anti-folate Cancer Diagnosis [Title/Abstract].

### Eligibility criteria

#### Inclusion criteria

Included RCTs met the minimum threshold dispensed by the Patient/Population, Intervention, Comparison, Outcomes, and Studies (10, 11).

The inclusion threshold in this systematic review was as follows:

I. Population: Studies with patients who have been subjected to the recently-developed aspects of one-carbon metabolism in cancer diagnosis, treatment and participants who reported drug resistance
II. Intervention: One-carbon metabolism
III. Comparison: Standard care or no care
IV. Outcomes: All forms of outcomes related to one-carbon metabolism in cancer diagnosis, treatment, and drug resistance
V. Studies: Randomized-controlled trials.

#### Exclusion criteria

Studies that did not meet the above-stated inclusion criteria were excluded from the review.

The exclusion criteria were as follows:

a) Non-randomized controlled trials (systematic reviews and meta-analyses, animal experiments, case studies, independent protocols, letters, and personal opinions).
b) Studies published in languages other than English.
c) Ongoing studies on the topic.

Studies investigating and reporting outcomes irrelevant to the present topic

### Evaluation of quality of literature

Studies that met the eligibility criteria were subjected to a quality evaluation in accordance with the Cochrane Risk of Bias (version 2.0) (12). The five domains applicable to the risk of bias assessments were fully pursued. That is random sequence generation, allocation concealment, blinding of participants and personnel, blinding of outcome assessment, incomplete outcome data, selective reporting, and other forms of bias. The five domains of risk of bias were assessed to determine the overall risk of bias (as endorsed by the Cochrane Risk of Bias protocol). Evaluating the role of one-carbon metabolism in cancer is key to cancer management clinical practices as it will inform the diagnosis and treatment (13).

### Data selection and extraction

Two independent reviewers were tasked with data extraction from eligible studies. Again, data selection was informed by the above-stated. The reviewers reviewed the studies’ titles and abstracts and analyzed full texts to establish each of the studies’ relevance. Differences and discrepancies between the independent reviewers would be amicably solved before they proceed with the data extraction activities. The indecent reviewers’ activities were mostly limited to identifying studies per the inclusion and exclusion criteria established above. Again, randomized controls reporting the features of intestinal flora in cancer patients and the effects of oral probiotics on immunity in chemotherapy for colorectal cancer were prioritized.

## Results

### Study selection

**Figure 5:**
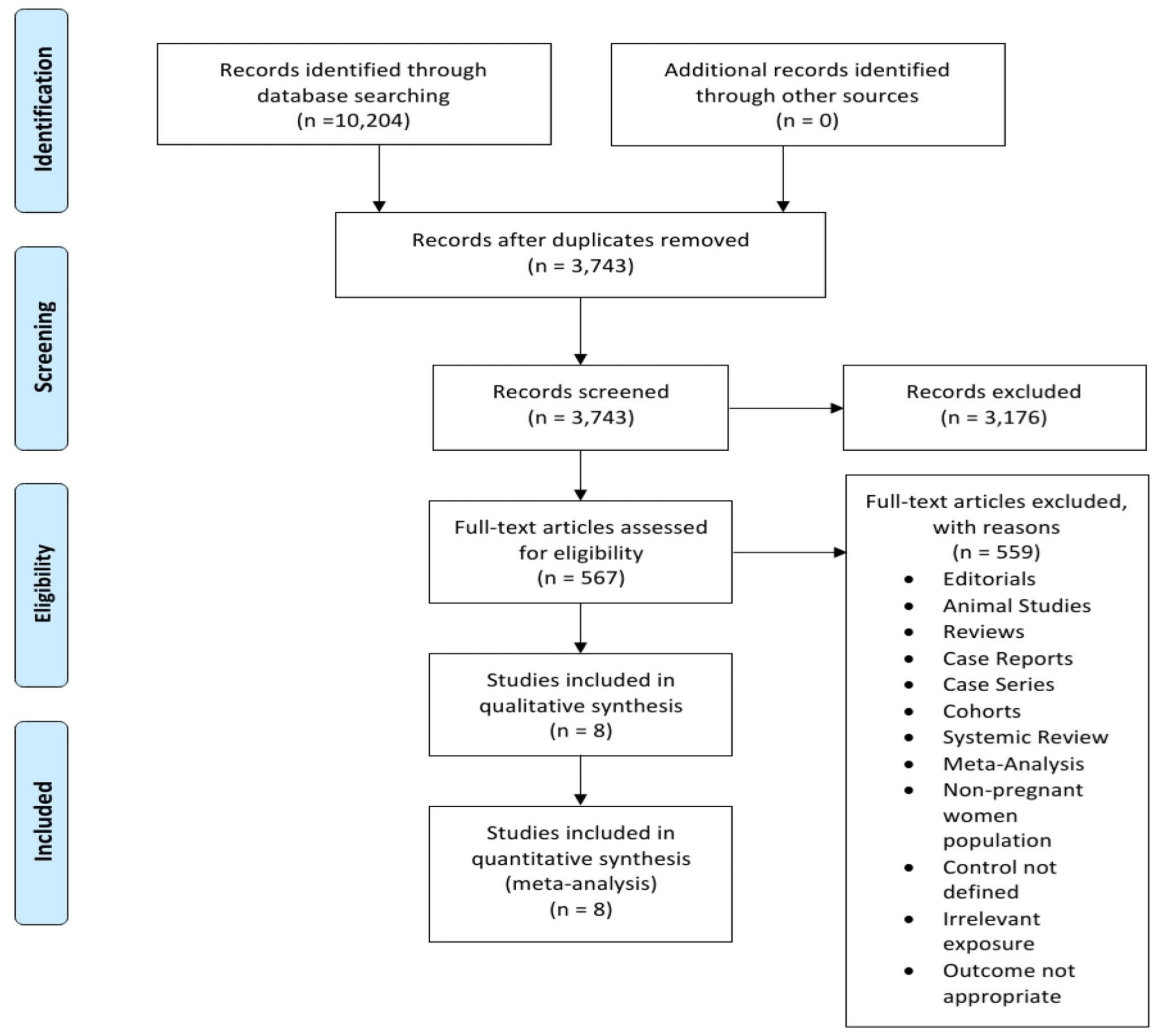
A Prisma flowchart diagram of the initial Literature search.

After a thorough literature search in the electronic databases, a total of 10,204 studies were identified out of 3,743 duplicates were removed. Another 3,743 records were screened, where 3,176 records were excluded, leaving 542 records set for full-text analysis for eligibility, out of which 567 studies were excluded for failing to meet the inclusion criteria. The excluded studies were non-randomized control trials, systematic reviews, meta-analyses, and editorials, studies published in languages other than English, studies involving animal specimens, case studies, and studies reporting outcomes irrelevant to the topic of discussion (**Figure 5**). Eight studies met the eligibility criteria and were included in the meta-analysis. Lastly, the literature search was limited to studies published from 1950 to the present. The initial literature search was performed per the PRISMA protocol. This guideline informed article identification, screening, and inclusion. **Figure 5** is a PRISMA flowchart illustrating study selection (literature search, study selection, screening, and inclusion), whereby the following studies met the inclusion threshold: (14), (7, 15–19, 6).

### Baseline characteristics of included RCTs

**Figure 6:**
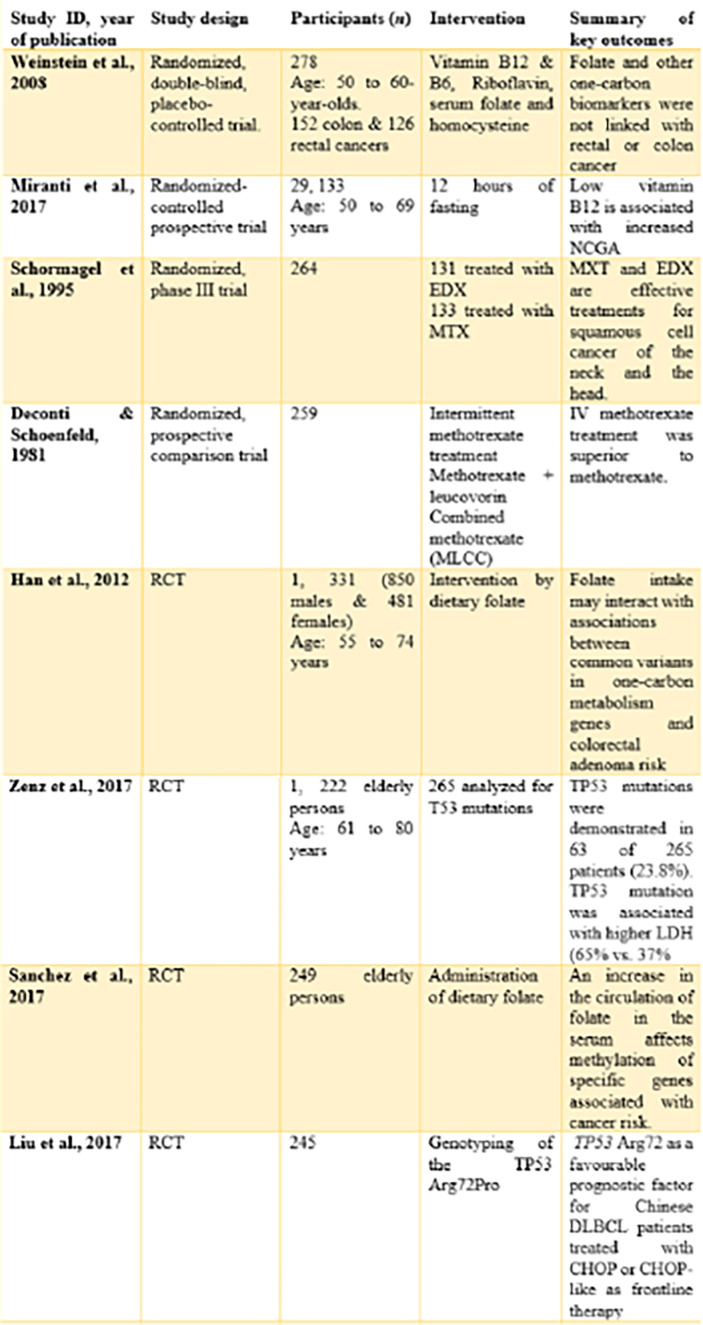
Characteristics of studies to be reviewed.

Eight randomized-controlled trials (with a total of 32,981 participants) were included in the review. These trials could be identified with unique features like origin, design, and investigated aspects related to the topic (**Figure 6**).

By origin, four trials (14), (7), (16), (17) originated from the United States of America. These studies were either performed or published in the United States of America.

One study (15) originated from the Netherlands. The study was carried out among the Dutch and published in the country.

One study (18) originated from Germany. The study was carried out within the German community and published in the country.

By design, all the included studies were randomized controlled trials. However, one study (14) is a randomized, double-blind, placebo-controlled trial. The study by (7, 15) was a randomized controlled prospective trial and a randomized phase III trial, respectively.

NCGA: Non-cardia gastric adenocarcinoma

EDX: Edatrexate

ADA: Adenosine deaminase

CD01: Cysteine dioxygenase

MTX: Methotrexate

MLCC: Cyclophosphamide and cytosine arabinoside

ML: Methotrexate with leucovorin rescue

### Quality appraisal

**Figure 7:**
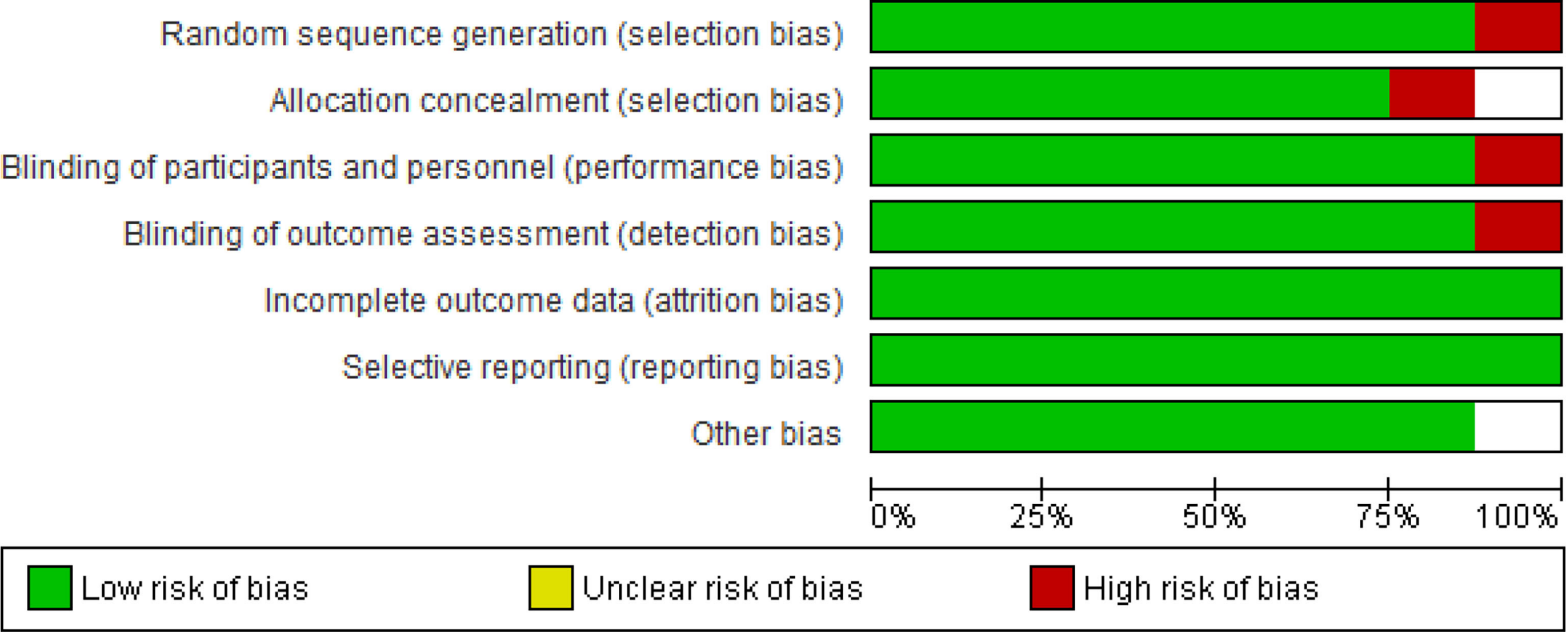
Risk of Bias graph: Review authors’ judgements about each Risk of Bias item presented as percentages across all included studies.

**Figure 8:**
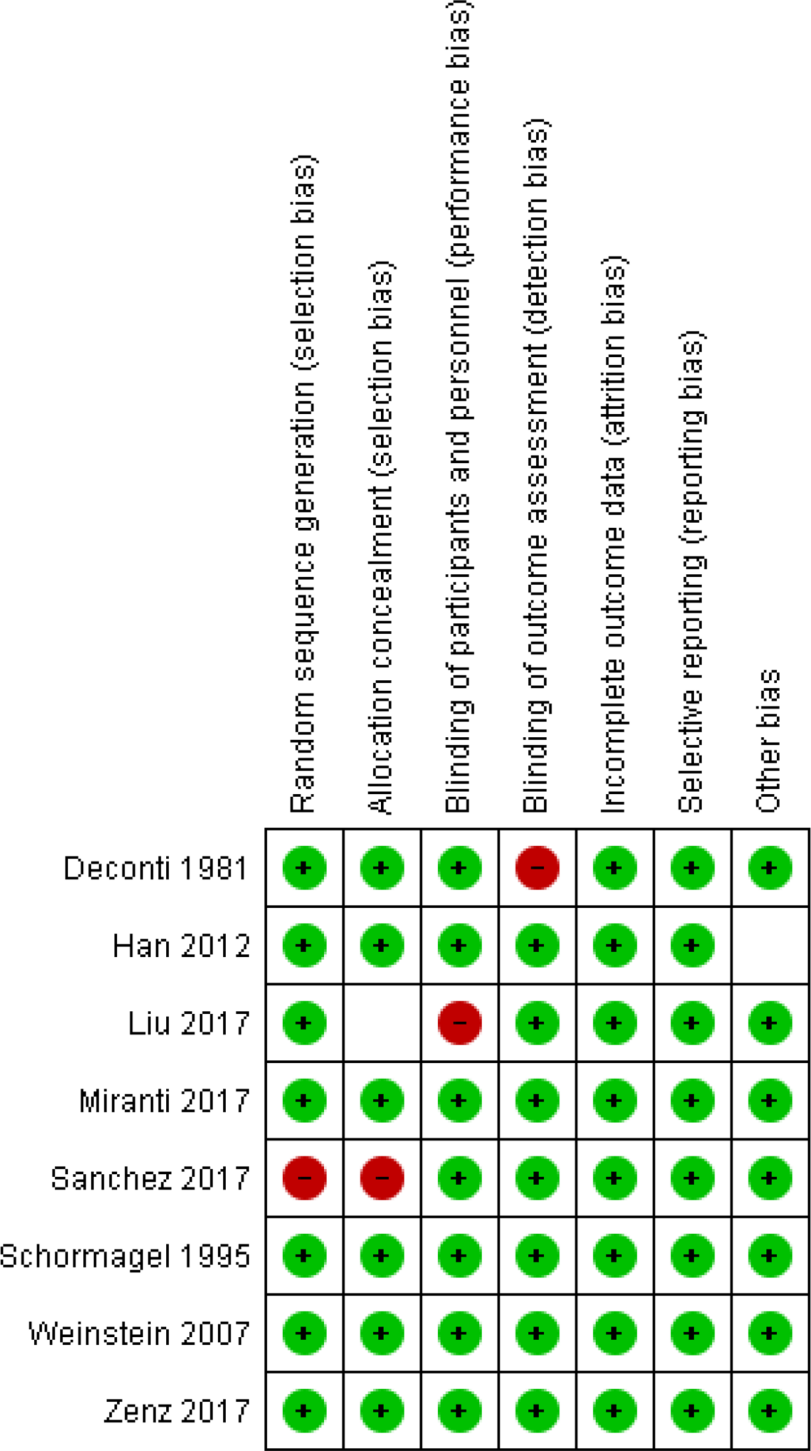
Risk of bias summary: Review authors’ judgements about each risk of Bias item for each included study.

Red parts represent the percentage of studies with a high overall risk of bias. While the green sections represent the percentage of studies with a low overall risk of bias, uncolored regions represent the percentage of studies found with a low overall unclear risk of bias (**Figure 7**). **Figure 8** represents the overall quality appraisal and the risk of bias assessment of individual studies.

### Quality assessments

The two investigators independently assessed the quality of each included study. The risk of biases from RCTs was assessed, through The Grading of Recommendations Assessment, Development, and Evaluation (GRADE) approach, in seven domains: Adequate Sequence Generation, Allocation Concealment, Blinding of Participants and Personnel, Blinding of Outcome Assessment, Incomplete Outcome Data, Selective Outcome Reporting and Other Bias (20). The individual domains and overall risk-of-bias judgment were expressed on three levels: low risk of bias, unclear risk of bias, and high risk of bias. Based on these factors, the overall quality of evidence was deemed low, moderate, or high risk of bias. This data has been entered in the summary of findings’ tables (GRADE details provided in supplemental files **Figure 8**).

### Highly active one-carbon metabolism and cancer diagnosis, treatment, and resistance

One study (14) reported the effect of one-carbon metabolism on colon and rectal cancer. This study would include folate intake and other one-carbon-related biomarkers with rectal or colon cancer. Weinstein et al. are categorical on vitamin B6’s crucial roles in inhibiting carcinogenesis in the colon.

One study (7) reported on cancer diagnosis and resistance as metabolic byproducts like vitamin B12 levels in the blood could be associated with cancer, particularly (NCGA). Mirant et al. found that vitamin B12 concentrations of at least 300 pmol/L can be used to diagnose non-cardia gastric adenocarcinoma (NCGA). At the same time, Mirant etal. (7) found that low folate levels in the blood can be used to diagnose esophagogastric junctional adenocarcinoma (EGJA).

One study (15) reported on the EDX and MTX’s effectiveness in the treatment of squamous cell cancer of the neck and the head (SCC). The two chemotherapeutic agents were moderately effective (15). However, MDX was found with enormous side effects, which incapacitated it as an indication of SCC.

One study (17) reported on stratification by the Adaptive Rank Truncated Product (ARTP) and dietary folate on gene-level and pathway links between genes related to one-carbon metabolisms and the identification of colorectal adenoma. The study concluded that the intake of folic acid could interact with the associations between the risk of colorectal adenoma and the genes related to one-carbon metabolism.

Two studies { (18, p. 1381-1388), (19, p. 1-7)} reported interesting accounts of highly reactive one-carbon metabolism on cancer resistance. A study by Zenz et al. reported that TP53 implicates negative prognostic impacts in the molecular group of diffuse large B-cell lymphoma (DLBCL). Diagnosis becomes an uphill task for this type of cancer with a poor prognosis.

Two studies (17, 6) reported on the outcomes of one-carbon metabolism on cancer mutation through the induction of dietary folate and vitamin B12. These study findings are key to cancer diagnosis, treatment, and resistance as they provide information on different aspects of one-carbon metabolism. Sanchez et al. reported that dietary vitamin B12 and folic acid could induce the methylation of the DNA, which is important for the early detection of cancers. DNA methylation and isolation are key to the early detection of cancers and resistance. In cancer treatment, high folate concentration induced methylation of the DNA responsible for the repair of cells (*p16, MGMT*, and *MLH1*). The study concluded that DNA methylation is the most studied epigenetic process and is considered the primary marker for early cancer detection. Sanchez et al.’s study corroborates Han et al.’s, where DNA synthesis, repair, and methylation were mediated by folic acid *via* the one-carbon metabolic pathways. In this study, folic acid mediated one-carbon metabolic pathways that altered genes that promoted the mutation of colorectal adenoma (17).

## Discussion

Folate metabolism, a critical step in the one-carbon metabolism cascade, is key to cancer diagnosis. Poor folate metabolism has been used to identify particular cancer types, especially prostate cancer (21). Different studies have reported divergent accounts of the interplay between folate metabolism and cancer diagnosis. Study outcomes by (14) did not report the link between cancer diagnosis and folate metabolism, as the latter was found not to affect the occurrence of colon and rectal cancers. Even though Weinstein et al.’s investigation does not point to a clear therapeutic or curative outcome, a prophylactic result is achieved through vitamin B6, which inhibits carcinogenesis in the colon. As opposed to Weinstein et al.’s findings, Miranti etal. (7) found that byproducts of one-carbon metabolism can be used to diagnose particular cancers and explain resistance effectively.

On EGJA diagnosis, Mirant et al.’s findings are consistent with one of the previous investigations (22) where low folate levels were used in diagnosis. Folate levels of (3.71 ± 0.30 ng/mL were used to diagnose gastric cancer (22). The discussion of cancer diagnosis and resistance takes the path of genes, as reported in two studies (17, 6). Folic acid and vitamin B12 mediate metabolic activities that induce genetic mutations that advance colorectal cancer (17) through one-carbon metabolism and trigger DNA methylation, which acts as an indicator for most types of cancer. In the latter’s case, early cancer detection becomes a hassle-free process, especially by measuring the levels of folic acid in the blood.

Even though highly active IC has been found with enormous benefits in cancer diagnosis and treatment, serious issues of concern remain. A study by (15) revealed that one-carbon metabolism resulted in side effects to the extent that the medication in question, MDX, was contraindicated against SCC. However, MDX and MTX were with moderate efficacy against SCC cancer (15). The use of methotrexate as an anti-tumor agent is key to understanding advances in IC therapy for cancer. MTX is an anti-folate that has been used as a treatment against acute lymphoblastic leukemia (ALL) (23). Recent advances show that it forms a wide array of regimens used in modern therapy for cancer (24–26).

Another study (16) reported a significant finding that precedes the advances in highly active carbon metabolism; a finding that supports the current claims by recent studies (15, 23–26). In this initial investigation, Deconti and Schoenfeld established that weakly administration of intravenous methotrexate stood as a superior intervention for epidermoid cancers of the neck and the head than on the combinations indicated for the same (ML and MLCC). These findings, when compared to Schornagel et al.’s investigation, demonstrate fundamental outcomes where the anti-folate, methotrexate, has been investigated and other indications have been established. To this point, MTX can be indicated for ALL and epidermoid cancers of the neck, confirming the expansion of the anti-folates in cancer management.

In cancer resistance, cancer cells have been reported to achieve unique adaptations that enable the survival of stress-related tumor growth and the satisfaction of anabolic proliferation requirements. Cellular metabolic activities like oxidative phosphorylation, glycolysis, anti-oxidant response, and glutaminolysis are influenced by the Tumor Suppressor Protein 53 (TP53), which, when compared to its role in mediating apoptosis throughout DNA-damaging stress, the TP53 also promotes cellular survival throughout metabolic stress: this process is key to tumor suppression and the performance of P53’s functions linked with non-cancers (27, p. 542-542). Study findings by Zenz et al. corroborate Maddock et al.’s position on T53’s role in mutations, alongside other roles in tumor suppression.

Zenz etal. (18), p. 1) found that TP53 mutates and implicates negative prognosis on DLBCL, which are important molecular markers in clinical management. Notably, these clinical markers inform precision-based medical intervention for aggressive lymphoma [through targeting B-cell Receptors (BCR), gene mutations, and alteration of pathways (28, p. 837), alongside the identifications of sub-groups who were unlikely to respond to R-CHOP].

TP53 has been found to enhance the survival of cancer patients. Particularly, TP53 is common with germinal center B-cell (GCB) and activated B-cell (ABC) sub-types, where it has been linked with high survival rates (18, p. 3). This mutation is key to the survival among individuals with primary central system lymphoma { (29, p. 5, 415), (30, p. 2, 071), (31, p. 530)}

A study by Liu et al. reported that TP53 Arg72 is a fundamental prognostic factor, particularly among the Chinese population. Further, the study reported that TP53 Arg72 did not influence the survival of DLBCL among European participants. Of this, advances in highly active 1C metabolism explored the health benefits of an intervention in a different ethnic group to establish outcomes and for comparative purposes. The findings from the Chinese population do not entirely rule out the importance of the intervention, as it can be used among the Chinese but not Europeans (32–34).

## Conclusion

Since the 1980s, remarkable advances have been made regarding the highly active 1C metabolism. This metabolic pathway can be used for cancer diagnosis, treatment, and explaining cancer resistance because different studies have produced strong evidence to point at the relationship between 1-carbon metabolism and cancer development.

A review of eight randomized controlled trials illustrated that the 1C metabolic pathway could mutate those genes and account for either tumorigenesis or treatment of cancers. At the same time, the diagnosis of various types of cancer is achieved by analyzing the concentrations or levels of metabolites like folic acid or vitamin B12/6. Notably, 1-carbon metabolism yields metabolites necessary for methylation, nucleotide synthesis, and reductive metabolism, which are key processes in the development of cancer cells. In clinical practice, detecting these metabolites could be a crucial confirmatory test for cancer. As for treatment, anti-folates could be key treatment interventions for cancer. I believe that one-carbon metabolism could be a game changer in carcer management through diagnosis and treatment approaches based on the metabolism of the one-carbon pathway. 

### Study limitations

• This is a major progression in learning as knowledge of various types of cancers is expanded. Conversely, accumulating knowledge on various types of cancers makes it impossible to make specific conclusions.
• Under-representation of the global community confines study outcomes to the specific communities where or among the population within which the study was performed. Participant selection was inadequate, as some of the studies focused on a particular ethnic community in the study. Again, a study by Liu et a. reported a previous finding with the European population, which ought to have been carried out again as the investigation on the Chinese participants was studied. Also, Sanchez et al. and Han et al. focused on elderly Chileans and Chinese participants, leaving out the rest of the population. Conclusions from these studies cannot apply to the general population, especially the young.

## Data availability statement

The original contributions presented in the study are included in the article/supplementary material. Further inquiries can be directed to the corresponding author.

## Author contributions

Y-XZ, SL,D-ML and Z-MW contributed to conception and design of the study. SL and Z-MW organized the database. SL wrote the first draft of the manuscript. Y-XZ, SL,D-ML and Z-MW wrote sections of the manuscript. All authors contributed to the article and approved the submitted version.

## Conflict of interest

The authors declare that the research was conducted in the absence of any commercial or financial relationships that could be construed as a potential conflict of interest.

## Publisher’s note

All claims expressed in this article are solely those of the authors and do not necessarily represent those of their affiliated organizations, or those of the publisher, the editors and the reviewers. Any product that may be evaluated in this article, or claim that may be made by its manufacturer, is not guaranteed or endorsed by the publisher.
